# Impact of COVID-19 lockdown restrictions on cardiac rehabilitation participation and behaviours in the United Kingdom

**DOI:** 10.1186/s13102-022-00459-5

**Published:** 2022-04-13

**Authors:** Richard Kirwan, Fatima Perez de Heredia, Deaglan McCullough, Tom Butler, Ian G. Davies

**Affiliations:** 1 School of Biological and Environmental Sciences, Liverpool John Moores University, Liverpool, UK; 2 Carnegie School of Sport, Leeds Beckett University, Leeds, UK; 3 Research Institute of Sport and Exercise Science, Liverpool John Moores University, Liverpool, UK; 4 Faculty of Health, Social Care and Medicine, Edge Hill University, Ormskirk, UK

**Keywords:** SARS-CoV-2, Physical activity, Cardiovascular disease, Coronary heart disease, Health behaviour, Exercise

## Abstract

**Background:**

COVID-19 lockdown measures led to the suspension of centre-based cardiac rehabilitation (CR). We aimed to describe the impact of lockdown on CR behaviours and perceptions of efficacy in a sample of CR participants.

**Methods:**

An online survey was conducted amongst CR participants from May to October 2020, COVID-19-related lockdown restrictions. Anthropometric data, participant-determined levels of motivation and self-perceived efficacy, CR practices etc., pre- and post-lockdown, were collected.

**Results:**

The probability of practicing CR in public gyms and hospitals decreased 15-fold (47.2% pre-, 5.6% post-lockdown; OR[95% CI] 0.065[0.013; 0.318], p < 0.001), and 34-fold (47.2% pre, 2.8% post; OR[95% CI] 0.029[0.004; 0.223], p < 0.001), respectively. Amongst participants, 79.5% indicated that their CR goals had changed and were 78% less likely to engage in CR for socialization after lockdown (47.2% pre, 16.7% post; OR[95% CI] 0.220[0.087; 0.555]; p = 0.002). The probability of receiving in-person supervision decreased by 90% (94.4% pre, 16.7% post; OR[95% CI] 0.011[0.002; 0.056]), while participants were almost 7 times more likely to use online supervision (11.1% pre, 44.4% post; OR[95% CI] 6.824[2.450; 19.002]) (both p < 0.001). Fifty percent indicated that their enjoyment of CR was lower than before lockdown and 27.8% reported they would be less likely to continue with CR in the newer format.

**Conclusions:**

Lockdown was associated with considerable changes in how CR was practiced, motivation levels and willingness to continue with CR. Further research is warranted to develop and improve strategies to implement in times when individuals cannot attend CR in person and not only during pandemics.

**Supplementary Information:**

The online version contains supplementary material available at 10.1186/s13102-022-00459-5.

## Background

Cardiovascular disease (CVD) is responsible for 1 in 4 deaths in the United Kingdom (UK) or over 170,000 deaths/year [1]. Cardiac rehabilitation (CR) is a primarily exercise-based intervention for those with established CVD or those at high risk of developing adverse cardiac events, aimed at reducing the risk of said events [2, 3]. Exercise-based CR is known to reduce the risk of mortality from CVD as well as the risk of cardiac-related hospitalisation, and also improving quality of life compared to non-exercise controls [2, 4, 5]. As such, exercise-based CR can be considered a key practice in maintaining the health of those at risk of CVD.

The COVID-19 pandemic developed into an unparalleled crisis leading to the execution of diverse measures aimed at reducing the spread of the virus. In the UK, travel bans, quarantine, isolation, and social distancing measures were enforced to varying degrees [6, 7]. This led to the suspension of group-based CR programmes, which are frequently hosted in hospitals, community centres and public gyms [8, 9] with up to 72% of Phase IV (long-term, community-based) CR programmes being suspended [10]. Long-term maintenance of physical activity, as observed in CR Phase IV, is known to lead to greater benefits to cardiac health [11]. Furthermore, these restrictions were observed to lead to decreases in physical activity (PA) and increases in sedentary behaviour [12, 13]. This extended period of reduced physical activity has the potential to impact the health of the general population [14–16] and especially those at high risk of CVD [17]. Furthermore, those with CVD are at greater risk of COVID-19 mortality [18], and in turn severe infection may lead to cardiovascular complications and myocardial injury, putting this population at even greater risk [19, 20].

Accordingly, health professionals and physical and rehabilitation medicine (PRM) specialists who play a vital role in the management of CR programmes [21] were recommended to provide home-based CR resources, in order to maintain this essential health service [8]. This resulted in the increased use of formats such as telephone and online-video to facilitate at-home CR [10]. It has been demonstrated that home-based CR can be at least as effective as centre-based CR in terms of outcomes such as blood pressure, total cholesterol, psychological status and exercise capacity [22, 23]. However, there is little data relating to the use of the particular modalities of at-home CR employed during the COVID-19 pandemic. As such, it is unknown whether these particular methods of at-home CR could negatively impact home-based CR, or result in reduced uptake, inadequate implementation and/or reduced efficacy. This may result from a number of obstacles including but not limited to lack of appropriate exercise equipment/facilities, inadequate supervision and poor motivation due to exercising alone [24].

Determining the impact of COVID-19 lockdown restrictions on CR-related exercise behaviours and PA, as well as motivation to continue with CR, may provide valuable information for improving current home-based CR recommendations. This may lead to more efficacious CR for those who are unable to or do not wish to participate in centre-based CR and may also be useful for rapid and effective implementation of CR in potential future waves of COVID-19 or other pandemics. The aim of this study was to determine the impact of COVID-19 lockdown restrictions on CR behaviours and perceptions of effectiveness, motivation and intent to continue.

## Methods

### Design

A cross-sectional, online questionnaire was conducted amongst CR participants, from May to October 2020, while the UK experienced varying degrees of COVID-19-related lockdown restrictions. Briefly, in March 2020, schools and non-essential businesses were closed, unnecessary travel was discouraged, time outside of the home was limited and social distancing guidelines were enforced. This included suspension of “non-essential” healthcare services such as CR. From May 2020, restrictions began to be gradually eased, while still encouraging social distancing measures. In October 2020, due to high rates of COVID cases, a “three-tier” COVID system was implemented based on area-specific infection rates [25].

### Recruitment

**Fig. 1:**
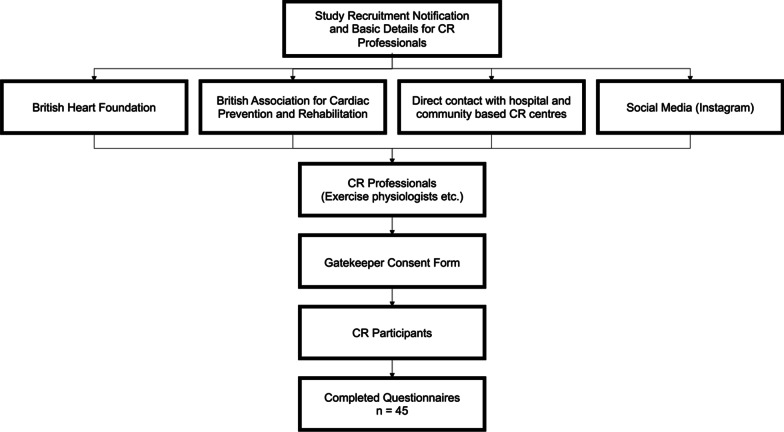
Recruitment flow diagram. *CR* cardiac rehabilitation

The questionnaire was distributed via a variety of methods including the authors’ interpersonal and professional networks, social media (Instagram), and through direct contact with centre-based CR services in the UK. Initial contact was with CR service providers, *i.e.*, CR-certified exercise physiologists and exercise instructors or related professional bodies such as British Association of Cardiac Prevention and Rehabilitation (BACPR) and British Heart Foundation (BHF). Instructors were asked to sign a gatekeeper consent form and then contact their CR participants directly using email and/or text messages including a link to the online questionnaire. The link included a participant information sheet with information on the study as well as a consent form and screening questionnaire. A recruitment flow diagram can be seen in Fig. 1.

### Ethics

This study received ethical approval from the University Research Ethics Committee at Liverpool John Moores University (UREC reference: 20/NSP/026). The study was conducted according to the ethical principles of the Declaration of Helsinki [26] and online informed consent was obtained from all participants before participation.

### Participants

A screening questionnaire was used to ensure participants fulfilled the following requirements: (1) they were participating in phase IV CR regularly at least twice per week before the UK Government implemented lockdown measures to limit the spread of COVID-19; (2) participant’s face-to-face CR was suspended due to Government regulations in response to the COVID-19 pandemic; (3) participant’s cardiac condition was not congenital or due to drug or alcohol abuse; (4) participants were older than 18 years; (5) participants were not pregnant. Phase IV CR was selected due to the significant impact of COVID-19 restrictions on the suspension of such programmes and the importance of long-term physical activity maintenance for improving cardiac health [10, 11].

### Implementation and assessments

The questionnaires were administered through JISC Online Surveys (Bristol, UK) and took approximately 40 min to complete. An ad hoc online questionnaire was developed to gather the following information: age, gender, area of residence, ethnicity, height and weight, as well as questions relating to CR practices and perceptions, pre- and post-lockdown. These included questions on CR location, exercise types, CR modality (group, online etc.), duration, frequency, perceived effort, perceived efficacy, goals, motivation etc.

The survey also included further, validated questionnaires relating to physical activity levels, quality of life, psychological-wellbeing, food access and perceptions around healthy eating. Data from these questionnaires will be discussed in separate publications.

### Data analysis

The demographic characteristics and the length of engagement with CR were compared between participants still actively engaged in their programmes and those who had quit CR after lockdown, by means of non-parametric Mann–Whitney tests. For those participants still conducting some form of CR, we compared their behaviours, attitudes, and conditions of CR practice before and after lockdown. Changes after lockdown in location of CR practice, goals, mode of CR practice/supervision, purchase of equipment, and moment of the day for CR practice were analysed by binomial regression, using a generalised linear model with repeated measures and fixed effects for time (post *vs.* pre-lockdown) and sex (women *vs.* men). Duration and frequency of the sessions were compared before and after lockdown by McNemar-Bowker tests (as these were categorical variables). Due to low counts, the initial categories for duration of exercise (*i.e.*, 0–20 min, 20–40 min, 40–60 min, and > 60 min/session) were pooled into two categories: 0–40 min and > 40 min/session, and frequencies were compared between before and after lockdown with the McNemar test. Number of exercises per session and participant’s change in perceived effort were analysed by repeated measures Wilcoxon signed ranked tests. All analyses were conducted with IBM SPSS software v. 26. Statistical significance was set at p < 0.05, and Bonferroni corrections were applied to account for multiple testing in the binomial regressions, according to the number of tests conducted for each variable (namely the number of different locations, goals, mode of practice, etc.).

## Results

### Demographics

A total of 45 people participated in the questionnaire. Demographic details of the sample are presented in Table 1. Thirty-six participants (80%) were still participating in some form of CR at the time of the study. There were trends for this subsample to have been engaged in their programme for longer and to be older, although these differences were not significant (U = 101.5, p = 0.086 and U = 103.5, p = 0.097, respectively). Similarly, other demographic variables (sex, ethnicity, or body mass index) were not significantly associated with the likelihood of continuing with or ceasing CR.

**Table 1:** Demographic characteristics of the sample

	All (n = 45)	Still engaging in CR (n = 36)	No longer engaging in CR (n = 9)
Age (years)	70 (63, 74)	71 (63.8, 74.8)	67 (61.5, 70.5)
Male (%)	88.9	91.7	77.8
White ethnicity (%)	91.1	88.9	100.0
Height (cm)	175.0 (169.0, 182.0)	176.5 (169.8, 182.3)	172.0 (162.5, 177.5)
Weight (kg)	79.3 (73.6, 92.6)	79.4 (73.0, 93.0)	77.1 (70.2, 92.4)
Body mass index (kg/m^2^)	25.4 (23.9, 29.4)	25.2 (23.8, 29.3)	28.5 (25.0, 29.7)
Time in CR before lockdown (weeks)	70.0 (22.5, 229.0)	102.0 (50.5, 257.5)	22.0 (8.5, 130.0)
Engaging in CR at time of study (%)	80	–	–

Values are presented as percentages, or as median (Q1, Q3). Participants still actively engaged in their CR programmes and those who had quit CR were compared by Mann–Whitney tests, significance set at p < 0.05; n for multiple testing

*CR* cardiac rehabilitation

### CR location before and after lockdown

**Fig. 2:**
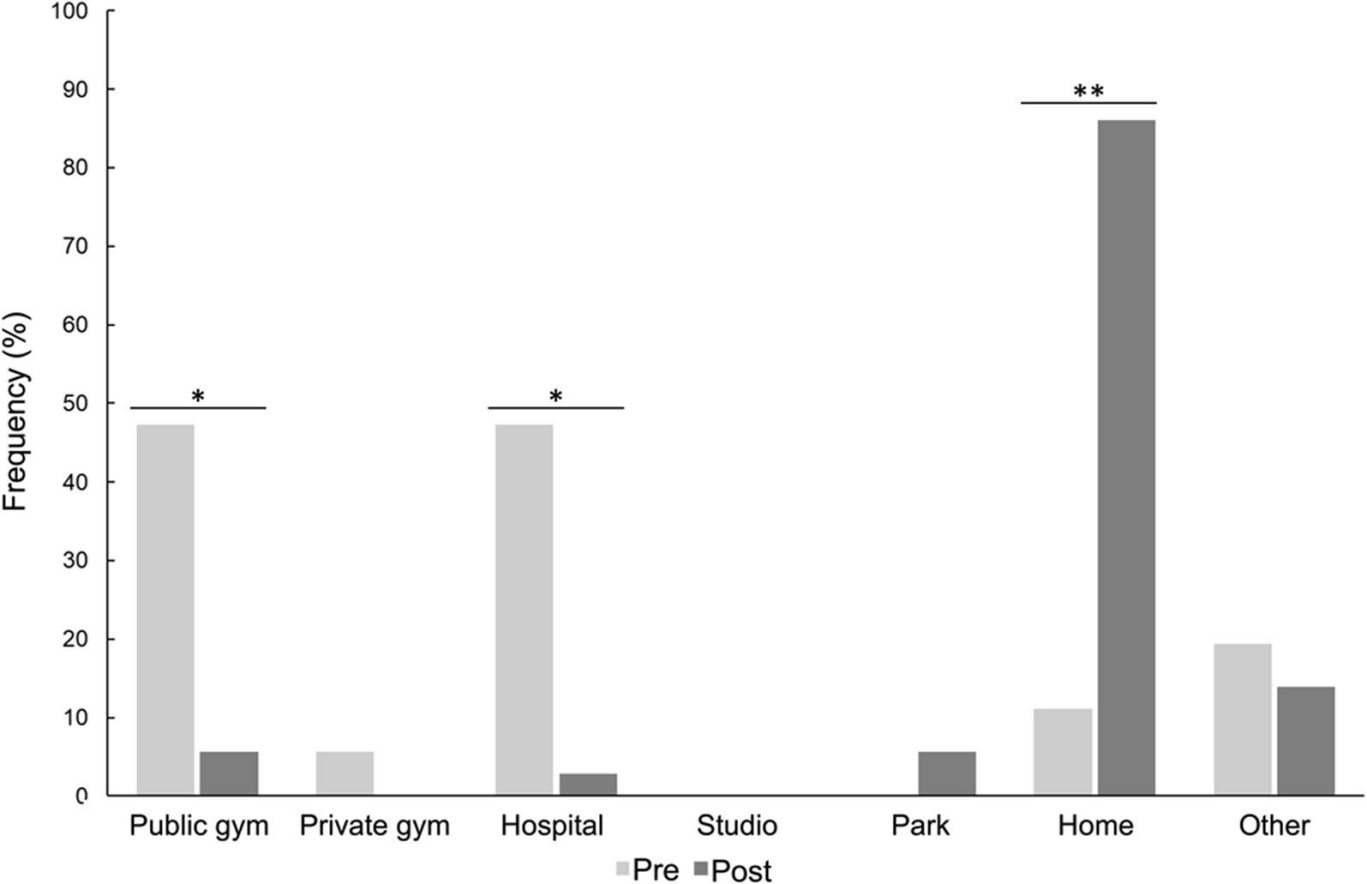
Influence of lockdown on location of cardiac rehabilitation practice. Bars represent the proportion (%) of participants who responded “Yes” to practising CR in each location. The change in the probability of practicing CR in a given location post-lockdown *vs.* pre-lockdown was analysed by mixed model GLM with repeated measures. *, ** Significant differences after post-hoc correction for multiple testing, *p < 0.0083; **p < 0.001

Compared to before lockdown, the probability of participants attending public gyms decreased 15-fold (47.2% pre- *vs.* 5.6% post-lockdown; OR [95% CI] 0.065 [0.013; 0.318]), and the probability of performing CR at hospitals fell by 34-fold (47.2% pre- *vs.* 2.8% post-lockdown; OR [95% CI] 0.029 [0.004; 0.223]) (both p < 0.001). In contrast, participants were 59 times more likely to engage in CR at home (11.1% pre- *vs.* 86.1% post-lockdown; OR [95% CI] 59.2 [14.4; 244.0]) (p < 0.001) (Fig. 2 and Additional file 1: Table S1). In general, women were significantly less likely to conduct CR at hospitals than men (OR [95% CI] 5.4 × 10^−5^ [2.7 × 10^−5^; 0.0]; p < 0.001) (Additional file 1: Table S1).

### Goals

**Fig. 3:**
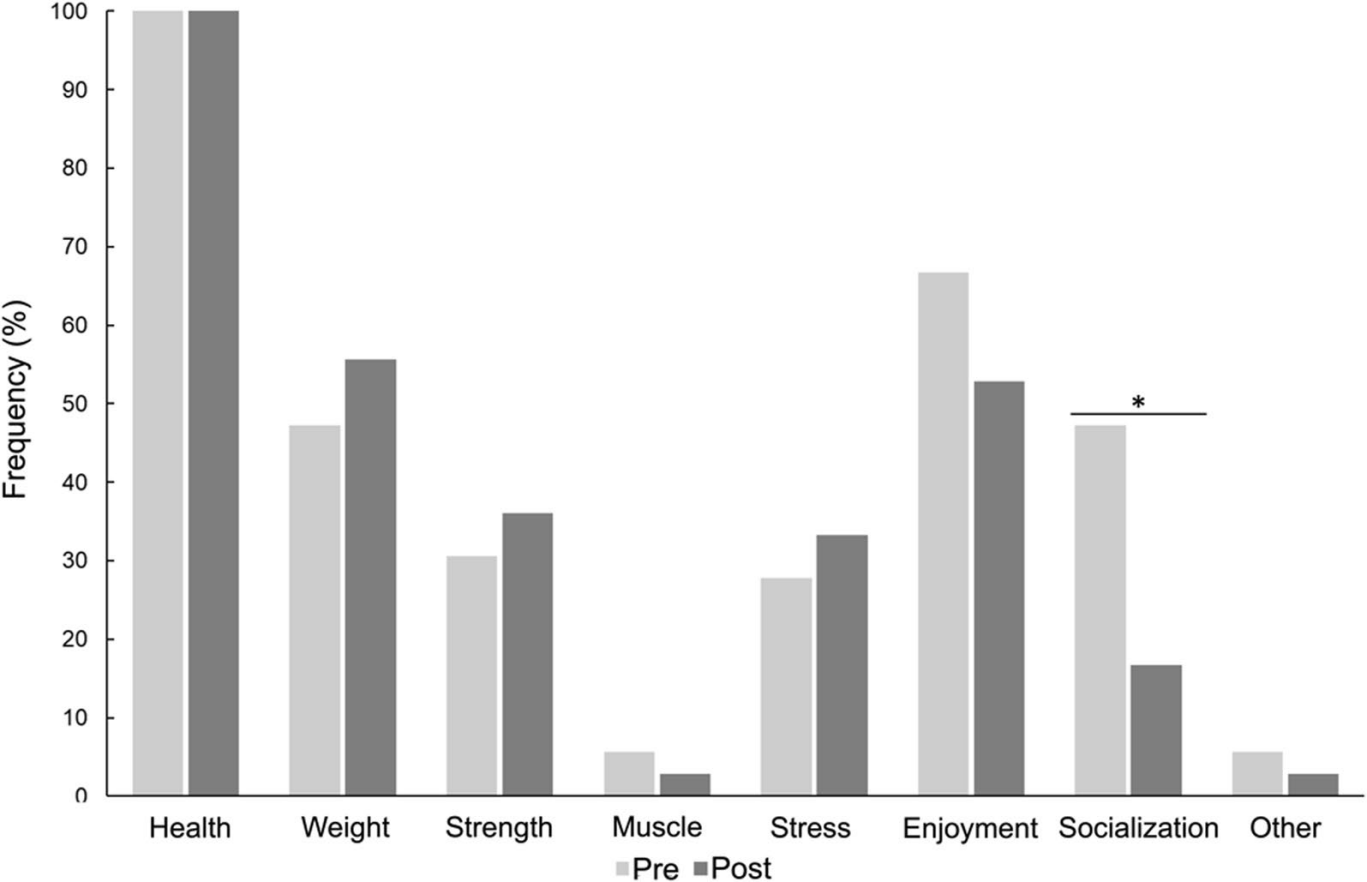
Influence of lockdown on cardiac rehabilitation-related goals. Bars represent the proportion (%) of participants who responded “Yes” to practising CR in each location. The change in the probability of practicing CR for a given goal post-lockdown *vs.* pre-lockdown was analysed by mixed model GLM with repeated measures. *Significant differences after post-hoc correction for multiple testing, *p < 0.0071

During lockdown, 79.5% of participants indicated that their CR goals had changed compared with pre-lockdown. Of note, participants were 78% less likely to engage in CR for socialization after lockdown (47.2% pre- *vs.* 16.7% post-lockdown; OR [95% CI] 0.220 [0.087; 0.555]; p = 0.002) (Fig. 3 and Additional file 1: Table S2). Regardless of lockdown, women were significantly more likely to conduct CR for increasing muscle mass (OR [95% CI] 1.7 × 10^7^ [1.0 × 10^6^; 2.7 × 10^8^]; p < 0.001) (Additional file 1: Table S2).

### Mode of practice/supervision and purchase of equipment

**Fig. 4:**
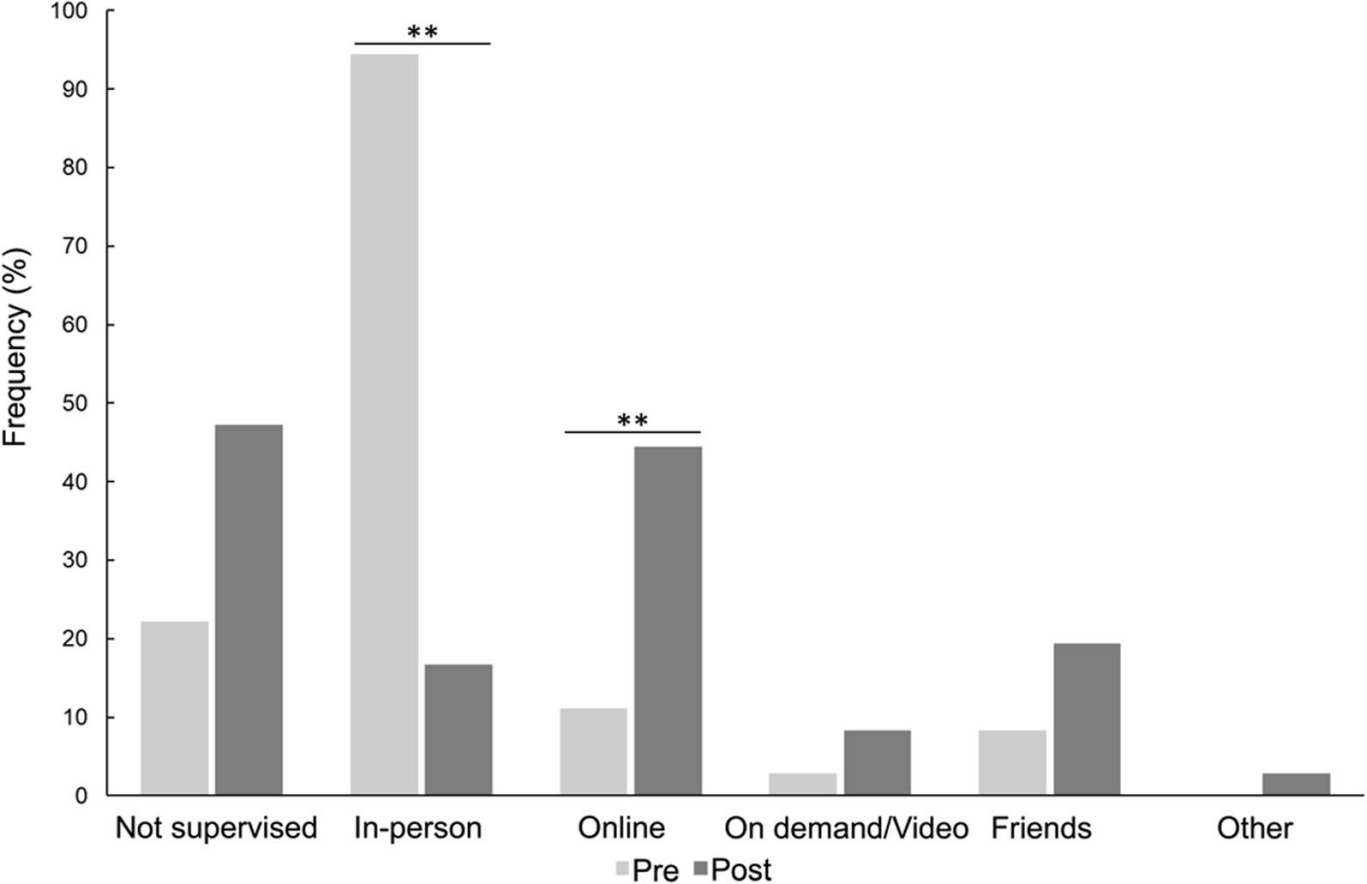
Influence of lockdown on cardiac rehabilitation mode of practice/supervision. Bars represent the proportion (%) of participants who responded “Yes” to practising CR in each location. The change in the probability of practicing CR in a given mode of supervision post-lockdown vs. pre-lockdown was analysed by mixed model GLM with repeated measures. *, **Significant differences after post-hoc correction for multiple testing, *p < 0.0083; **p < 0.001

There were significant changes in the supervisory arrangements after lockdown. The probability of receiving in-person supervision decreased by 90% (94.4% pre- vs. 16.7% post-lockdown; OR [95% CI] 0.011 [0.002; 0.056]), while participants were almost 7 times more likely to use online supervision (11.1% pre- vs. 44.4% post-lockdown; OR [95% CI] 6.824 [2.450; 19.002]) (both p < 0.001), compared with pre-lockdown (Fig. 4). Women were significantly less likely to engage in online and on-demand/video supervision (online OR [95% CI] 2.76 × 10^−5^ [1.41 × 10^−5^; 5.40 × 10^−5^]; on demand/video OR [95% CI] 0.00 [6.56 × 10^−5^; 0.00]; both p < 0.001) (Additional file 1: Table S3).

Prior to lockdown, 25% of participants had purchased specific equipment (including weights, machines, bands and/or other devices) to support their CR, while 36.1% participants indicated they had purchased specific equipment since the implementation of lockdown measures. From the latter, 30.8% had also answered “Yes” to purchasing equipment prior to lockdown. Half of the participants did not purchase equipment either before or after lockdown. COVID-19 restrictions did not seem to have an influence on the purchase of exercise equipment (Additional file 1: Table S3).

### Time of day

The time of day when participants conducted CR changed significantly after the lockdown, with participants shifting from evening practice (4.7 times less likely compared to pre-lockdown: 58.3% pre- vs. 25.0% post-lockdown; OR [95% CI] 0.214 [0.098; 0.467]; p < 0.001) towards morning practice (3.7 times more likely: 30.6% pre- vs. 61.1% post-lockdown; OR [95% CI] 3.663 [1.542; 8.700]; p = 0.004) (Additional file 1: Fig. S1 and Additional file 1: Table S4). Regardless of restrictions, women were significantly less likely to conduct CR later in the day (evening and night) than men (evening OR [95% CI] 1.13 × 10^−5^ [5.77 × 10^−6^; 2.20 × 10^−5^; night OR [95% CI] 0.002 [0.000; 0.016]; both p < 0.001) (Additional file 1: Table S4).

### Number, duration, and frequency of CR exercises

There was no difference in the number of exercises per session after lockdown (W = 80.0, p = 0.074) (Additional file 1: Fig. S2), but the duration of the sessions increased significantly: prior to lockdown, only 22% of participants reported to exercise for more than 40 min/session, and this increased to 72% after lockdown (Χ^2^ = 13.136, p < 0.001) (Additional file 1: Fig. S2). Finally, there was a trend for participants to shift towards the lowest (once per week) or the highest exercise frequencies (more than 3 times/week) but this was not statistically significant (Χ^2^ = 9.200, p = 0.056) (Additional file 1: Fig. S3).

### Perceived effort, motivation, enjoyment, and willingness to continue with cardiac rehab

**Fig. 5:**
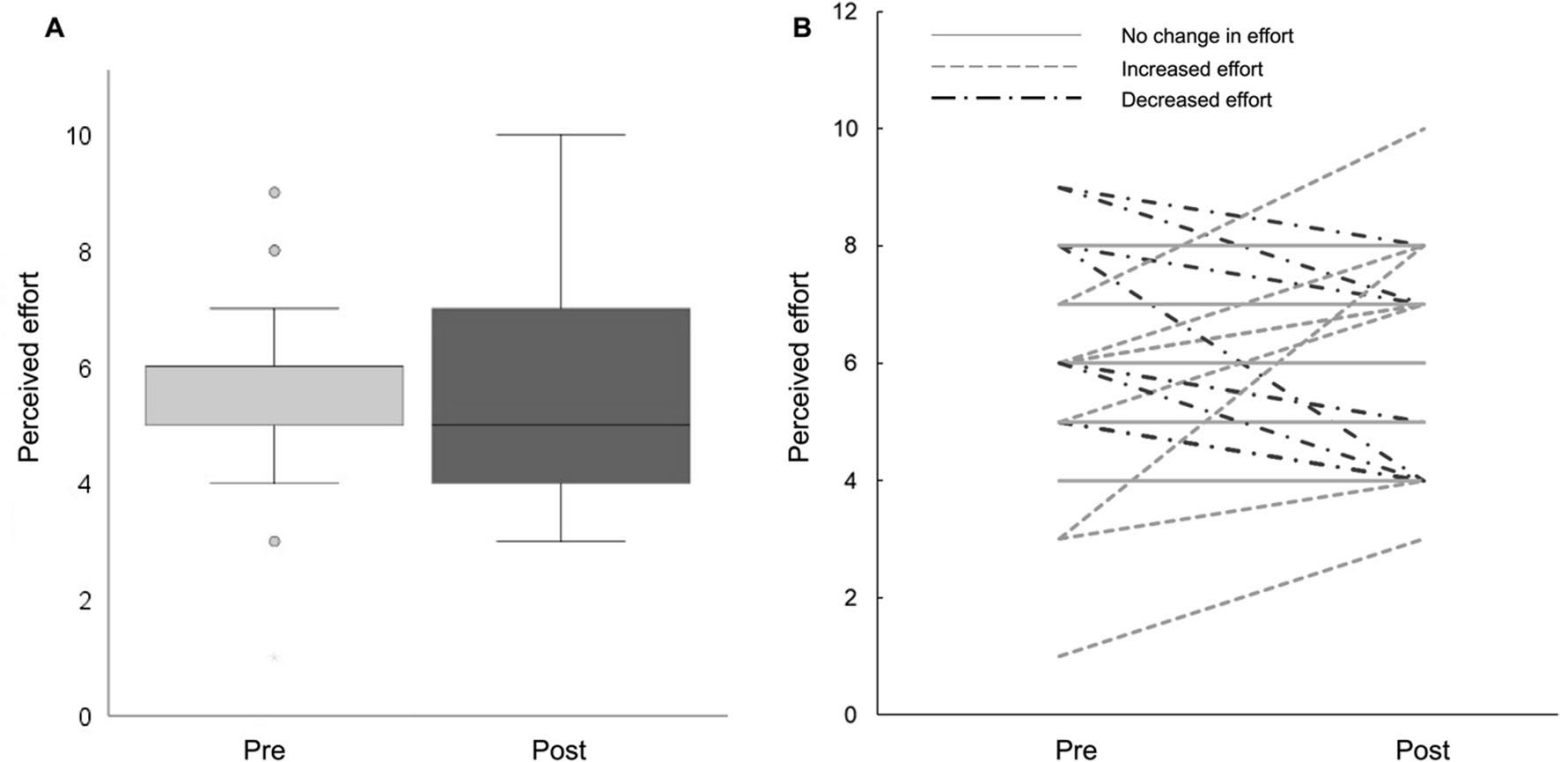
**a** Influence of lockdown on perceived effort of cardiac rehabilitation in our sample. Differences between pre- and post-lockdown were analysed by repeated measures Wilcoxon signed ranked tests, p < 0.05. **b** Individual changes in perception of effort. Simple dashed lines indicate increased perceived effort post-lockdown; dot-dashed lines indicate decreased perceived effort post-lockdown; solid lines indicate no-change in perceived effort post-lockdown

Overall participants’ perception of effort increased significantly during lockdown (median [Q1; Q3] 5 [4; 7], *vs.* pre-lockdown 6 [5; 6]; W = 335.5; p < 0.001) (Fig. 5a), although with considerable heterogeneity, with 66.7% participants reporting an increase, 25% reporting a decrease and 9% reporting similar levels of perceived effort (Fig. 5b).

**Fig. 6:**
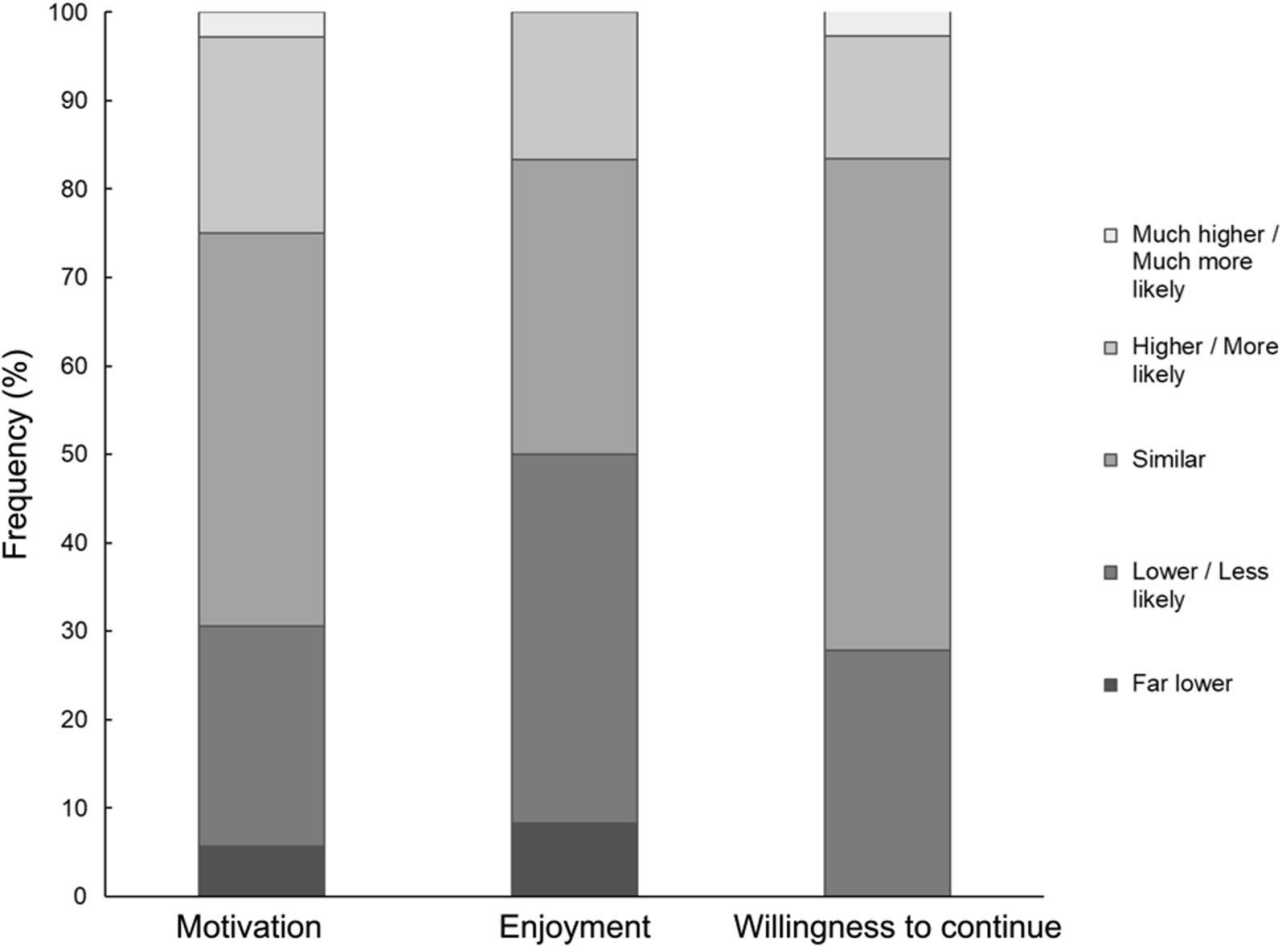
Motivation to practice, enjoyment of and willingness to continue with cardiac rehabilitation in comparison with pre-lockdown levels

Changes in motivation levels were symmetrical, with most participants (44.4%) experiencing similar levels of motivation pre- and post-lockdown, and comparable proportions of participants experiencing either more or less motivation (Fig. 6a). However, 50% of participants indicated that their enjoyment of CR was either lower or far lower than before lockdown, and only 16.7% indicated they enjoyed CR more after lockdown (Fig. 6b). Despite this, compared with pre-lockdown, only 27.8% of participants reported they would be less likely to continue with CR in the post-lockdown format, 55.6% admitted they would be similarly likely to continue with CR, and 16.7% were more likely or much more likely to continue (Fig. 6c).

## Discussion

In this study we aimed to determine the impact of COVID-19 lockdown restrictions on CR behaviours and perceptions of effectiveness, motivation and intent to continue. Our results indicate that COVID-19 lockdown restrictions were associated with decreased participation in CR, changes in CR location, goals, supervision, duration and enjoyment, and increased perceived effort. While most participants were willing to continue with CR in its COVID-modified form, almost 30% indicated they were less likely to do so.

Physical activity is an essential component of a healthy lifestyle with higher levels of physical activity associated with reduced risk of CVD, type-2 diabetes mellitus (T2DM), sarcopenia, osteoporosis, cognitive decline and depression [27–32]. In cardiac populations, physical activity in the form of CR has been shown to reduce the risk of future cardiac events and mortality [2, 4, 5]. As such, the importance of maintaining CR has been highlighted during the COVID-19 pandemic due to suspension of some CR services [33]. It is understood that the benefits of exercise for cardiac health are most significant when exercise is performed continuously and in the long-term [11] as research has indicated that the benefits of CR may be lost within as little as 3 months of CR cessation [34]. While there are no established guidelines for the frequency or duration of CR, current UK guidelines recommend individuals accumulate 150 min of moderate intensity activity or 75 min of vigorous activity per week, while minimizing sedentary behaviour [35]. Highlighting a potential reduction in physical activity levels, data from the present study revealed there was a trend for an increase in the number of individuals participating in CR only once per week during lockdown (p = 0.056).

The establishment and maintenance of regular routines is regarded as a key determinant of whether individuals maintain a health-oriented behaviour, such as exercise [36]. While poorly studied, interference with regular routines and habits may reduce the likelihood of those exercise habits being maintained in the long-term, thus reducing the likelihood of health benefits being attained and maintained [37]. Accordingly, the occurrence of the COVID-19 pandemic and the associated restrictions on movement and social gatherings may potentially have detrimental effects on maintenance of effective CR exercise habits or routines. Indeed, a recently published BACPR survey of healthcare professionals revealed that as many as 72% of Phase IV CR services were suspended during the COVID-19 pandemic, with almost half of respondents indicating they were no longer providing services for high-risk patients [10]. Furthermore, the onset of COVID-19 lockdown restrictions were reported to reduce activity levels, in the form of step counts, by 16% in a population of heart failure patients [38]. Furthermore, research from CR participants in Japan revealed that COVID-19 related interruptions in CR practice were negatively associated with both activity levels and hemodynamic response, relative perceived exertion during exercise as well as body weight [39]. Older patients in particular (≥ 75 years), were also at greater risk of frailty [39].

The maintenance of motivation for CR activities should be considered vitally important for their long-term success. While initial behaviour change motivations amongst CR participants may be rooted in the fear of uncertainty regarding their long term health, it has been hypothesized that motivation for maintaining such behaviours may be different [40]. Temporal self-regulation theory suggests that the enjoyment of such new behaviours may encourage individuals to maintain their practice in the long-term [41]. Apart from enjoyment, the satisfaction of other psychological needs is known to encourage behaviour maintenance and has been reported to be predictive of future home based exercise in CR populations [42]. Accordingly, promoting the fulfilment of psychological needs and enjoyment of CR should be viewed as important for its own maintenance. In this study, 50% of participants indicated that their enjoyment of CR was either lower or much lower than pre-lockdown levels. While only 27.8% of participants reported they would be less likely to continue with CR in its current format, these are still concerning results and highlight the potential importance of guidance aimed at making home-based CR more enjoyable.

CR participants may also be influenced by the perception of attaining results from their exercise efforts [40]. Self-efficacy theory posits that positive perception of one’s results may reinforce motivation and encourage individuals to maintain their efforts, a theory that has support in the field of exercise maintenance [43]. While personal accomplishments are important for self-efficacy, external factors including vicarious experience (observing peers achieve success with an endeavour) and verbal persuasion (verbal cues and feedback, leading to a belief in one’s ability to succeed) also play a major role [44]. These vicarious experiences and verbal persuasion are often key features of in-person exercise classes and as such, contact with peers and CR exercise providers/trainers can help encourage self-efficacy [45, 46] and potentially exercise maintenance.

Of particular concern, a recent BACPR survey of CR healthcare professionals highlighted that the three most widely used “technologies” for delivery of CR during the pandemic were telephone, pre-recorded video, and email [10]. While these technologies played an important role in allowing CR services to continue operating [47], little is known about their efficacy, particularly in encouraging maintenance of CR through the fulfilment of psychological needs and enjoyment. It also remains to be seen how these methods compare in efficacy to other less frequently employed technologies such as live-video conferencing and smart device applications, also identified in the BACPR survey [10]. It should also be mentioned that the same survey highlighted multiple barriers to the adoption of new technologies amongst both healthcare professionals and patients including lack of patient confidence, lack of patient access to internet and suitable devices such as computers, and professionals lack of confidence in using technology to deliver CR services [10]. Further research into the potentially efficacy of different technologies for delivering CR, as well as research into overcoming the barriers that may inhibit its use by healthcare professionals and patients is warranted.

The results of this study highlight the importance of preparing and implementing strategies to provide not only adequate but also engaging CR when individuals cannot attend CR sessions in person. Such strategies may be of benefit not only to those who cannot attend CR due to pandemic restrictions but also due to issues such as, logistical difficulties in attending CR sessions, geographical isolation, unwillingness to attend group CR etc. This may help augment the uptake of CR which currently has global uptakes of 10–60% [48, 49], and which has been designated as a goal of the UK Department of Health [50].

If non-centre-based CR is to be promoted and developed, a pertinent question is how effective are such home-based CR interventions? Centre-based CR has been shown to be more effective (improved physical endurance and lower serum risk factors such as total and low-density lipoprotein cholesterol) compared to control groups which only received recommendations to exercise [51]. However, a Cochrane systematic review and meta-analysis comparing centre-based with home-based CR found them both to be equally effective in terms of mortality risk, risk of cardiac events, exercise capacity, modifiable risk factors and health related quality of life [23]. This discrepancy may be explained by many of the home-based interventions incorporating clearly planned exercise routines as well as regular support in the form of telephone calls or visits from CR staff [52]. Promising strategies to incorporate into home-based CR include distance services such as telehealth which can include both education and supervised exercise delivered via telephone, video-conferencing and mobile apps [53, 54]. Indeed the use of mobile apps has been shown by a recent meta-analysis to improve CR adherence by up to 1.4 times that of controls [55], while CR approaches incorporating virtual reality and videogames can improve motivation and adherence, and increase physical activity [56].

### Limitations

There are a number of limitations to our present study. Firstly, recruitment proved difficult and resulted in a low sample size (n = 45). The low recruitment rates were potentially due to two factors: (i) the multiple steps required to contact participants i.e., initially contacting CR-organizations in order to disseminate news of the study to CR-certified exercise physiologists and exercise instructors, requiring gate-keeper consent, and subsequent gatekeeper requirements to contact potential participants by asking them to forward an email with the questionnaire link. The complexity and multiple steps of this process may have resulted in lower likelihood of uptake; (ii) the exclusion criteria, which formed part of the initial screener questionnaire were possibly too strict as they were intended to recruit suitable participants for a longitudinal investigation. This resulted in the exclusion of participants that, for example, were in CR Phase 3, were participating in CR less than 2 × per week, had their face-to-face CR suspended in response to the COVID-19 pandemic and/or whose cardiac condition was congenital or due to drug or alcohol abuse. As such, the population used in our study cannot be considered representative of the UK CR population in general. Future, cross-sectional investigations of CR could benefit from including such individuals in their recruitment efforts and screen-out later, if necessary.

Furthermore, the number of women recruited to this study was low at 11.1%. This number is considerably lower than the average percentage of female CR participants in England, Wales and Northern Ireland, which ranges from 27.8 to 31.3% [57]. We cannot speculate as to the reasons for the low level of female participation in our study but as our population was predominantly male, the results may not be applicable to female CR participants. The ethnicity of our study population was predominantly white (British/Irish/Other white) at 91.1%, which is similar to a recently published report of CR demographics that reported 83.8% of participants as white [57]. As such, the findings of this report may not be applicable to ethnic minorities participating in CR and clearly more efforts are needed to capture insights from these groups.

## Conclusions

Lockdown was associated with considerable changes in how CR was practiced, levels of motivation and importantly, willingness to continue with this activity. Further research is warranted to develop and improve strategies to implement in times when individuals cannot attend CR in person and not only during pandemics.

## Supplementary Information

**Additional file 1**. Supplementary material.

## Data Availability

The data underlying this article will be shared on reasonable request to the corresponding author.
