# Supplementary material for: Impact of COVID-19 lockdown restrictions on cardiac rehabilitation participation and behaviours in the United Kingdom
Source: BMC Sports Sci Med Rehabil. 2022 Apr 13;14:67. doi: 10.1186/s13102-022-00459-5 (PMC9007266; doi:10.1186/s13102-022-00459-5)
Supplement: Supplementary file 1 — Additional file 1. Supplementary material. [file 13102_2022_459_MOESM1_ESM.docx]

**Impact of COVID-19 lockdown restrictions on cardiac rehabilitation participation and behaviours in the United Kingdom**

**Authors**

Richard Kirwan, School of Biological and Environmental Sciences, Liverpool John Moores University, Liverpool, UK. <https://orcid.org/0000-0003-4645-0077>

Dr Fatima Perez de Heredia, School of Biological and Environmental Sciences, Liverpool John Moores University, Liverpool, UK. (Joint corresponding author) <https://orcid.org/0000-0002-2537-3327>

Dr Deaglan McCullough, Carnegie School of Sport, Leeds Beckett University, Leeds, UK. Research Institute of Sport and Exercise Science, Liverpool John Moores University, Liverpool, UK. (Joint corresponding author) <https://orcid.org/0000-0002-9882-9639>

Dr Tom Butler, Faculty of Health, Social Care and Medicine, Edge Hill University, Ormskirk, UK. <https://orcid.org/0000-0003-0818-1566>

Dr Ian G. Davies, Research Institute of Sport and Exercise Science, Liverpool John Moores University, Liverpool, UK. <https://orcid.org/0000-0003-3722-8466>

**Running Head**

Cardiac rehab practices during COVID restrictions

**Corresponding authors**

Dr Deaglan McCullough, Carnegie School of Sport, Leeds Beckett University, Leeds, UK. Research Institute of Sport and Exercise Science, Liverpool John Moores University, Liverpool, UK. [D.McCullough@leedsbeckett.ac.uk](mailto:D.McCullough@leedsbeckett.ac.uk)

Dr Fatima Perez de Heredia, School of Biological and Environmental Sciences, Liverpool John Moores University, Liverpool, UK. [F.PerezDeHerediaBenedicte@ljmu.ac.uk](mailto:F.PerezDeHerediaBenedicte@ljmu.ac.uk)

# Supplementary material


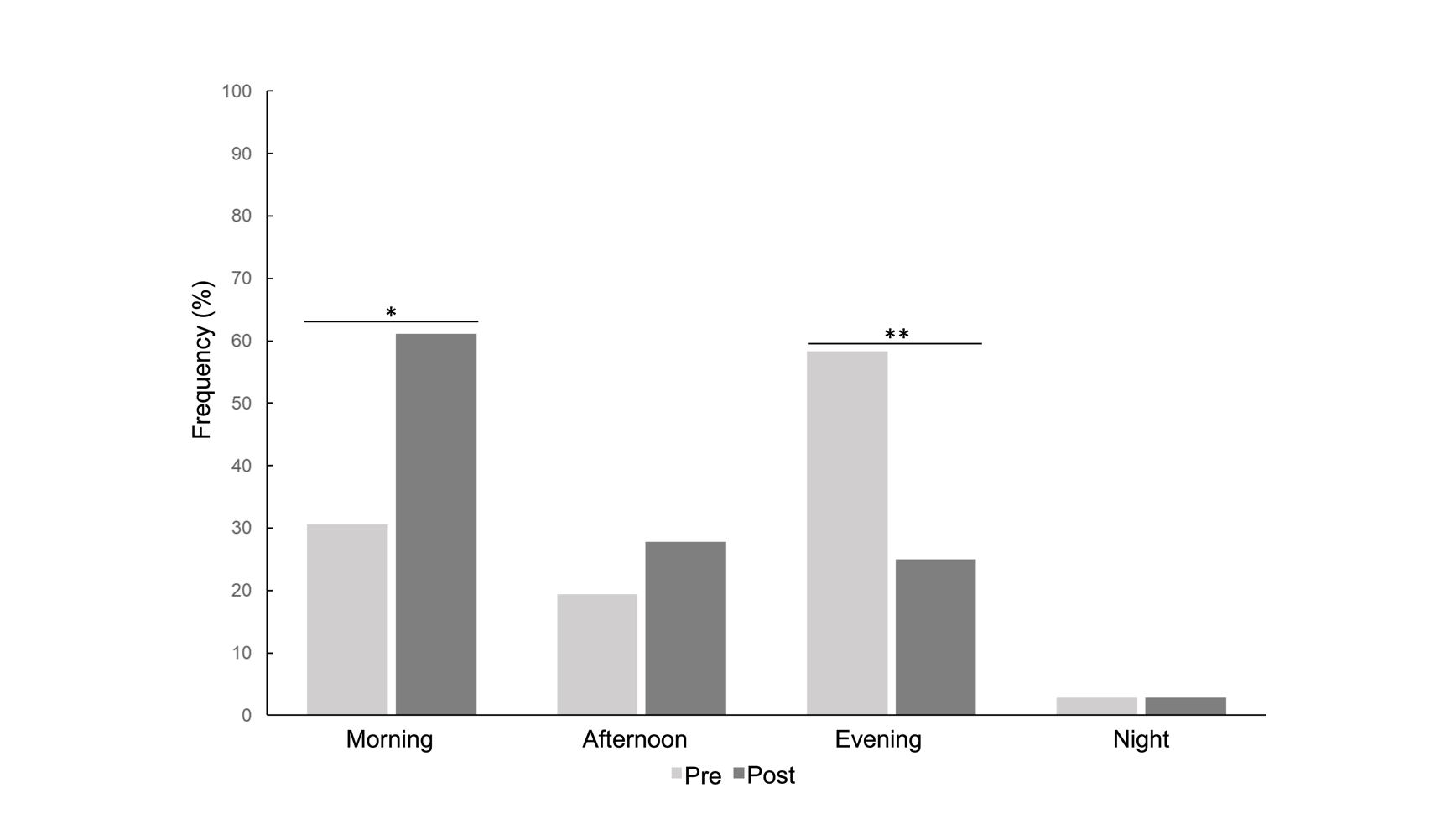


Supplementary Figure 1. Influence of lockdown on cardiac rehabilitation practice time. The change in the probability of practicing CR at a given time of day post-lockdown *vs.* pre-lockdown was analysed by mixed model GLM with repeated measures. ^(^*^,^ **^)^ Significant differences after post-hoc correction for multiple testing, *p<0.0125, **p<0.001.

# Supplementary material


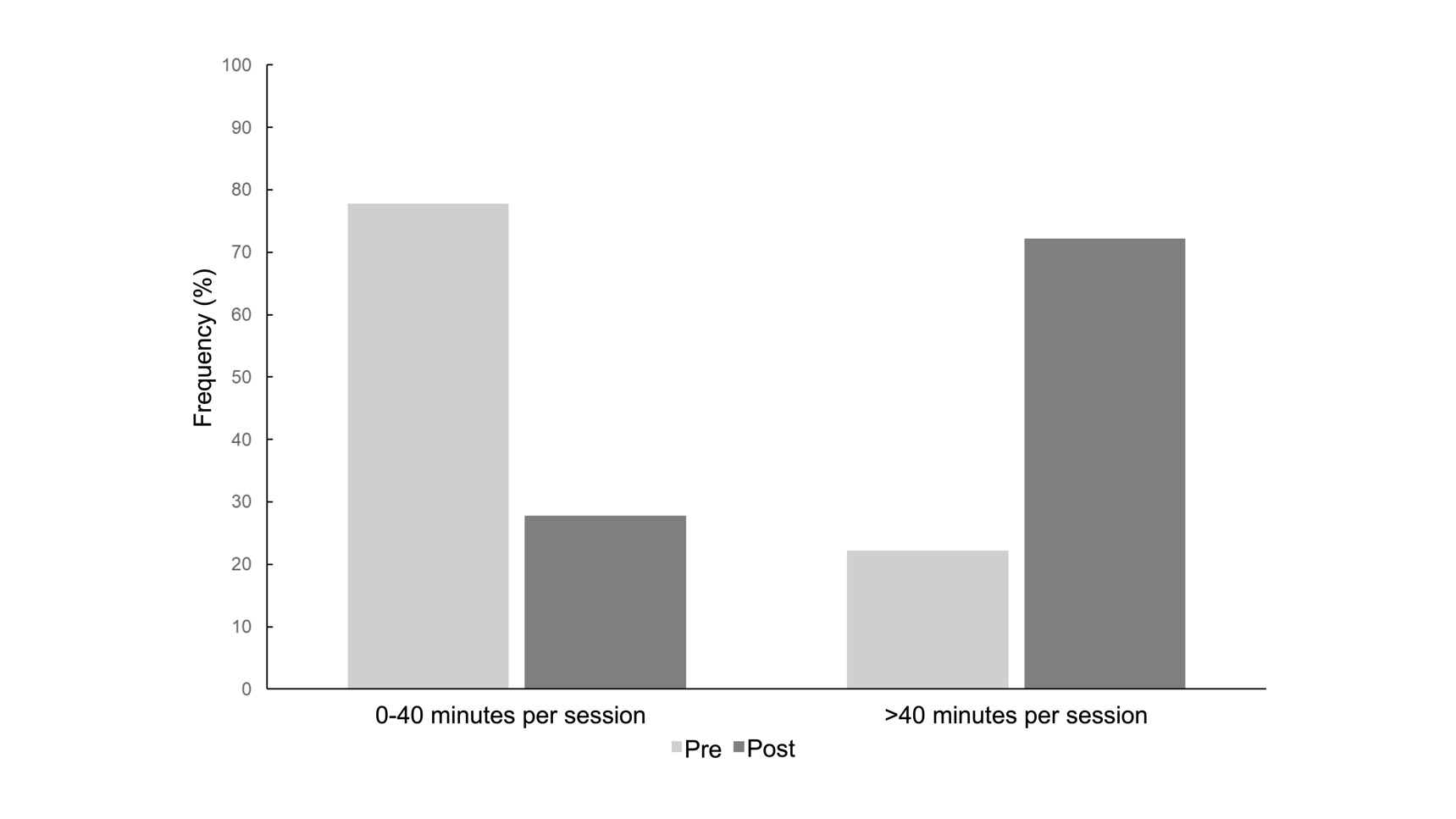


Supplementary Figure 2. Influence of lockdown on cardiac rehabilitation duration. The proportion of participants who reported to exercise for more than 40 min/session increased from 22% to 72% after lockdown (Χ2=13.136, p<0.001, assessed by McNemar test).

# Supplementary material


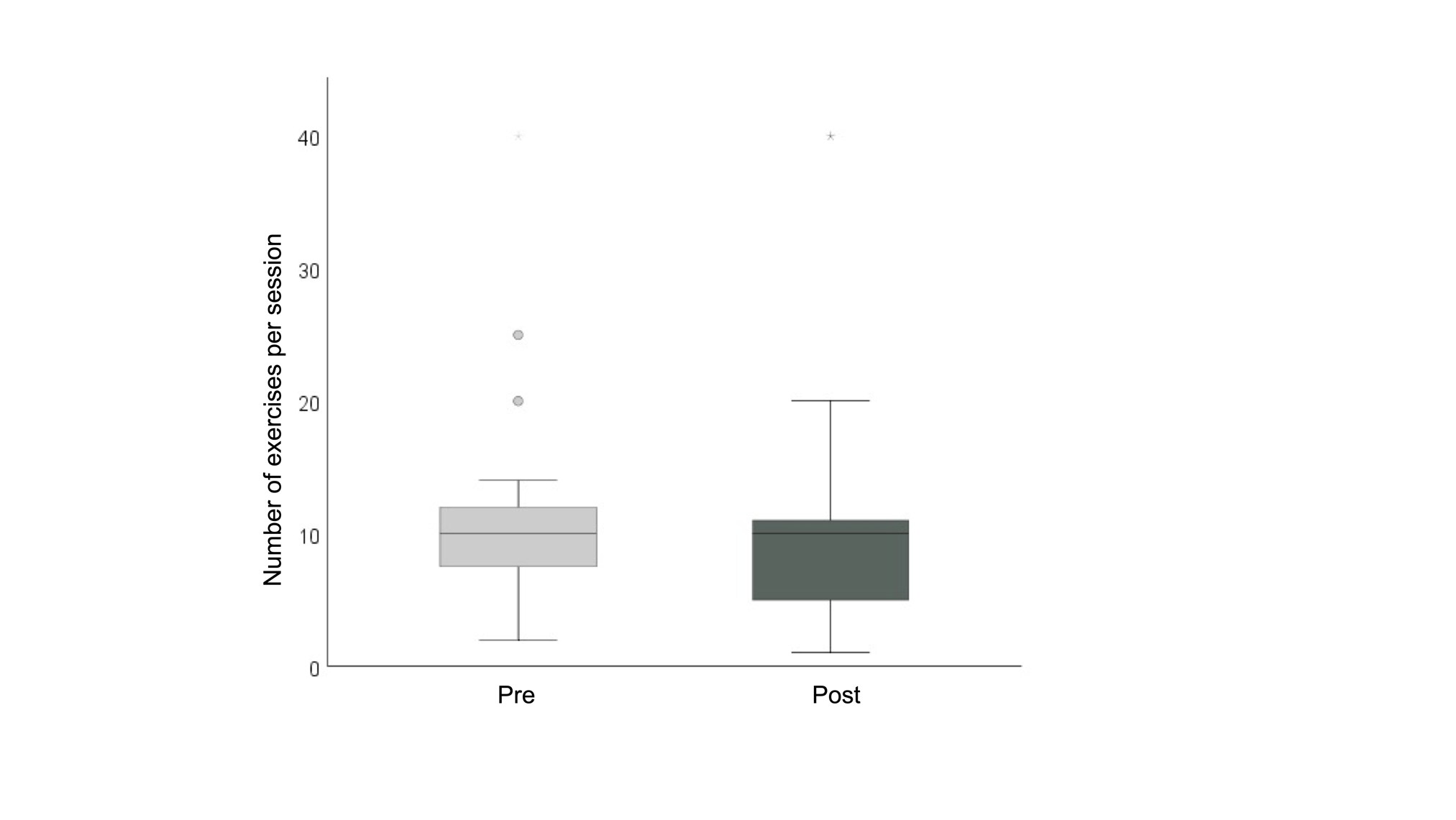


Supplementary Figure 3. Influence of lockdown on number of cardiac rehabilitation exercises used per session. There was no difference in the number of exercises per session before and after lockdown (W=80.0, p=0.074, assessed by Wilcoxon signed ranked test).

# Supplementary material


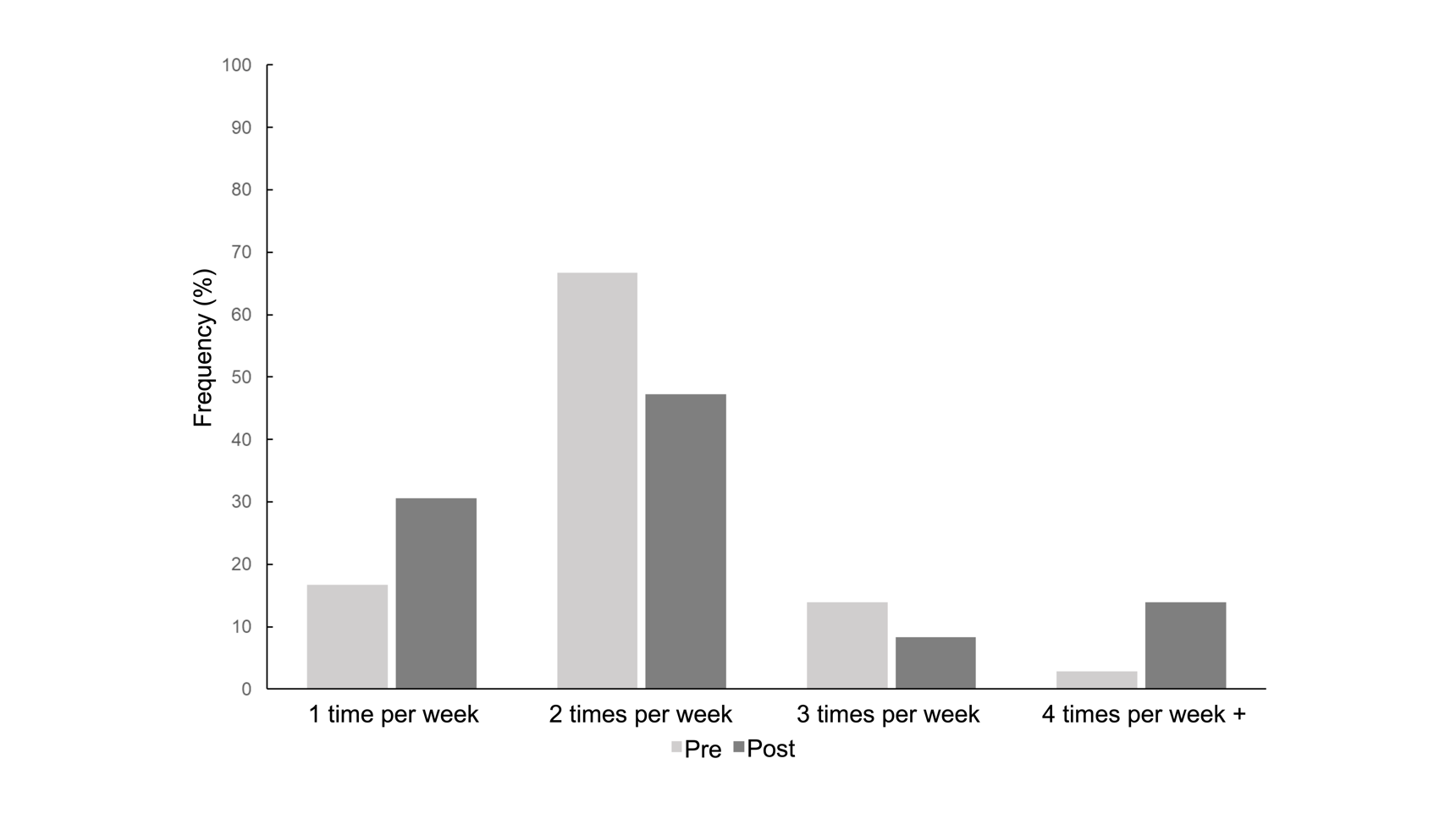


Supplementary Figure 4. Influence of lockdown on weekly frequency of cardiac rehabilitation. Most participants exercised twice per week, with a trend to shift towards lower (once per week) or higher exercise frequencies (more than 3 times/week), but this change was not statistically significant (Χ2=9.200, p=0.056, assessed by McNemar-Bowker test).

# Supplementary material

Supplementary Table 1. Influence of lockdown on CR location.

|  | **Post v Pre-lockdown** | | | **Women v Men** | | | **Constant** | | **Model** | |
| --- | --- | --- | --- | --- | --- | --- | --- | --- | --- | --- |
|  | Β (SE) | OR  (95% CI) | P | Β (SE) | OR  (95% CI) | P | Β (SE) | P | % Correct  classification | P |
| **Public gym** | **-2.736 (0.7971)** | **0.065**  **(0.013; 0.318)** | **0.001** | -0.779 (1.1732) | 0.459  (0.044; 4.764) | 0.509 | **3.565 (1.2875)** | **0.007** | **73.6** | **0.003** |
| **Private gym** | -0.843 (0.6340) | 0.430  (0.122; 1.525) | 0.188 | -4.533×10^-7^  (6.2127×10^-7^) | 1.000  (1.000; 1.000) | 0.468 | 3.520 (5.7103×10^-7^) | <0.001 | 97.2 | 0.188 |
| **Hospital** | **-3.526 (1.0155)** | **0.029**  **(0.004; 0.223)** | **0.001** | **-9.828 (0.3445)** | **5.395×10^-5^**  **(2.714×10^-5^; 0.000)** | **<0.001** | **13.293 (0.9857)** | **<0.001** | **76.4** | **<0.001** |
| **Park** | 0.843 (0.6340) | 2.323  (0.656; 8.230) | 0.188 | -4.533×10^-7^  (6.2127×10^-7^) | 1.000  (1.000; 1.000) | 0.468 | 2.677 (0.6340) | <0.001 | 97.2 | 0.188 |
| **Home** | **4.081 (0.7096)** | **59.224**  **(14.377; 243.961)** | **<0.001** | 1.786 (1.1468) | 5.965  (0.605; 58.766) | 0.124 | **-3.529 (1.1679)** | **0.004** | **87.5** | **<0.001** |
| **Other** | -0.414 (0.7230) | 0.661  (0.156; 2.797) | 0.569 | 1.191 (0.7313) | 3.291  (0.765; 14.155) | 0.108 | 0.775 | 0.266 | 83.3 | 0.268 |

Mixed model: generalised linear model with repeated measures and fixed effects (time [post v pre-lockdown] and gender [women v men]). Values in bold are significant predictors for p<0.0083, after *post-hoc* correction for multiple testing.

# Supplementary material

Supplementary Table 2. Influence of lockdown on CR goals.

|  | **Post v Pre** | | | **Women v Men** | | | **Constant** | | **Model** | |
| --- | --- | --- | --- | --- | --- | --- | --- | --- | --- | --- |
|  | Β (SE) | OR  (95% CI) | P | Β (SE) | OR  (95% CI) | P | Β (SE) | P | % Correct  classification | P |
| **Weight management** | 0.334 (0.2441) | 1.397  (0.859; 2.273) | 0.175 | -0.062 (1.0047) | 0.940  (0.127; 6.977) | 0.951 | -0.167 (0.9572) | 0.862 | 54.2 | 0.395 |
| **Strength** | 0.281 (0.3413) | 1.324  (0.670; 2.617) | 0.413 | 2.635 (1.0183) | 13.946  (1.829; 106.338) | 0.012 | -1.860 (0.9258) | 0.048 | 72.2 | 0.014 |
| **Muscle growth** | -1.386 (3.0000) | 0.250  (0.001; 99.335) | 0.645 | **16.634 (1.4007)** | **1.676×10^7^**  **(1.025×10^6^; 2.740×10^8^)** | **<0.001** | **0.693 (1.2247)** | **0.573** | **91.7** | **<0.001** |
| **Stress** | 0.262 (0.2595) | 1.300  (0.775; 2.182) | 0.315 | 0.141 (1.2788) | 1.151  (0.090; 14.755) | 0.913 | 0.565 (1.2312) | 0.648 | 69.4 | 0.597 |
| **Enjoyment** | -0.583 (0.2457) | 0.558  (0.342; 0.911) | 0.020 | 0.333 (1.2916) | 1.395  (0.106; 18.346) | 0.797 | -0.417 (1.2442) | 0.739 | 59.7 | 0.066 |
| **Socialization** | **-1.515 (0.4643)** | **0.220**  **(0.087; 0.555)** | **0.002** | -1.030 (1.0792) | 0.357  (0.041; 3.075) | 0.343 | 2.578 (1.0455) | 0.016 | **68.1** | **0.003** |
| **Other goals** | -0.725 (0.7295) | 0.484  (0.113; 2.076) | 0.324 | **-8.435 (0.7608)** | **0.000**  **(4.758×10^-5^; 0.001)** | **<0.001** | **11.901 (0.489)** | **<0.001** | **95.8** | **<0.001** |

Mixed model: generalised linear model with repeated measures and fixed effects (time [post v pre-lockdown] and gender [women v men]). Values in bold are significant predictors, for p<0.0071, after *post-hoc* correction for multiple testing.

# Supplementary material

Supplementary Table 3. Influence of lockdown on mode of practice and supervision of CR, and on purchase of equipment.

|  | **Post v Pre** | | | **Women v Men** | | | **Constant** | | **Model** | |
| --- | --- | --- | --- | --- | --- | --- | --- | --- | --- | --- |
|  | Β (SE) | OR  (95% CI) | P | Β (SE) | OR  (95% CI) | P | Β (SE) | P | % Correct  classification | P |
| **Not supervised** | 1.154 (0.4440) | 3.171  (1.307; 7.688) | 0.011 | 0.750 (1.0695) | 2.117  (0.251; 17.881) | 0.485 | -0.577 (1.0472) | 0.583 | 66.7 | 0.035 |
| **In person** | **-4.511 (0.8183)** | **0.011**  **(0.002; 0.056)** | **<0.001** | 1.140 (1.1707) | 3.126  (0.302; 32.305) | 0.334 | 0.598 (1.0954) | 0.587 | **88.9** | **<0.001** |
| **Online** | **1.920 (0.5134)** | **6.824**  **(2.450; 19.002)** | **<0.001** | **-10.497 (0.3357)** | **2.76×10^-5^**  **(1.41×10^-5^; 5.40×10^-5^)** | **<0.001** | **10.558 (0.0669)** | **<0.001** | **72.2** | **<0.001** |
| **On demand/video** | 1.163 (1.2110) | 3.200  (0.286; 35.841) | 0.340 | **-8.618 (0.5085)** | **0.000**  **(6.556×10^-5^; 0.000)** | **<0.001** | **10.921 (0.2917)** | **<0.001** | **94.4** | **<0.001** |
| **Friends** | 1.018 (0.6525) | 2.766  (0.753; 10.168) | 0.123 | 1.502 (0.8617) | 4.492  (0.805; 25.060) | 0.086 | 0.092 (0.8003) | 0.909 | 86.1 | 0.160 |
| **Equipment purchased** | 0.533 (0.5385) | 1.705  (0.582; 4.994) | 0.326 | -0.955 (1.1931) | 0.385  (0.036; 4.168) | 0.426 | 1.458 (1.1790) | 0.221 | 69.4 | 0.455 |

Mixed model: generalised linear model with repeated measures and fixed effects (time [post v pre-lockdown] and gender [women v men]). Values in bold are significant predictors, for p<0.0083, after *post-hoc* correction for multiple testing.

# Supplementary material

Supplementary Table 4. Influence of lockdown on moment of the day to practice CR.

|  | **Post v Pre** | | | **Women v Men** | | | **Constant** | | **Model** | |
| --- | --- | --- | --- | --- | --- | --- | --- | --- | --- | --- |
|  | Β (SE) | OR  (95% CI) | P | Β (SE) | OR  (95% CI) | P | Β (SE) | P | % Correct  classification | P |
| **Morning** | **1.298 (0.4337)** | **3.663**  **(1.542; 8.700)** | **0.004** | 1.056 (1.3768) | 2.874  (0.184; 44.807) | 0.446 | -1.433 (1.3939) | 0.307 | 66.7 | 0.014 |
| **Afternoon** | 0.468 (0.4085) | 1.597  (0.707; 3.608) | 0.256 | 0.539 (1.2880) | 1.714  (0.131; 22.377) | 0.677 | 0.466 (1.2448) | 0.709 | 76.4 | 0.473 |
| **Evening** | **-1.540 (0.3909)** | **0.214**  **(0.098; 0.467)** | **<0.001** | **-11.395 (0.3352)** | **1.126×10^-5^**  **(5.767×10^-6^; 2.197×10^-5^)** | **<0.001** | **12.375 (0.3208)** | **<0.001** | **70.8** | **<0.001** |
| **Night** | **-5.297×10^-15^**  **(1.095×10^-14^)** | 1.000  (1.000; 1.000) | 0.630 | **-6.100 (1.0155)** | **0.002**  **(0.000; 0.017)** | **<0.001** | 9.566 (0.0000) | - | **97.2** | **<0.001** |

Mixed model: generalised linear model with repeated measures and fixed effects (time [post v pre-lockdown] and gender [women v men]). Values in bold are significant predictors, for p<0.0125, after *post-hoc* correction for multiple testing
